# Assessing the effects of diabetes mellitus on the monocyte-to-lymphocyte ratio and the QuantiFERON-TB gold plus assays for tuberculosis treatment monitoring: a prospective cohort study

**DOI:** 10.3389/fimmu.2024.1451046

**Published:** 2025-01-17

**Authors:** Paulo Ranaivomanana, Arimanitra Razafimahefa, Mame Ndiaye, Crisca Razafimahatratra, Haja Ramamonjisoa, Perlinot Herindrainy, Mamy Raherison, Antso Hasina Raherinandrasana, Julio Rakotonirina, Jonathan Hoffmann, Rila Ratovoson, Niaina Rakotosamimanana

**Affiliations:** ^1^ Mycobacteriology Unit, Institut Pasteur de Madagascar, Antananarivo, Madagascar; ^2^ Epidemiology and Clinical Research Unit, Institut Pasteur de Madagascar, Antananarivo, Madagascar; ^3^ Department of Diabetology, Association Malgache Contre le Diabète (AMADIA), Antananarivo, Madagascar; ^4^ Centre Hospitalier Universitaire de Soins et Santé Publique Analakely (CHUSSPA), Antananarivo, Madagascar; ^5^ Medical and Expertise Department, Fondation Mérieux, Lyon, France

**Keywords:** tuberculosis, treatment monitoring, diabetes mellitus, QuantiFERON-TB gold plus, white blood cell count, monocyte to lymphocyte ratio

## Abstract

Diabetes mellitus (DM) is an important risk factor for the development of active tuberculosis (TB). QuantiFERON-TB Gold Plus (QFT-P), white blood cell count (WBC) assays and monocyte-to-lymphocyte ratio (MLR) reflect the inflammatory reactions associated with TB and offer the potential to monitor TB treatment to allow a better management of the disease. The aim of this study was to assess the influence of DM on the respective performances of QFT-P and WBC assays in their capacities to monitor the treatment of drug-sensitive pulmonary TB (TBP). The QFT-P and WBC were prospectively compared between TB patients with and without DM at inclusion (D0), at the end of treatment (M6) and two months after the end of treatment (M8). After laboratory measurement of glycated hemoglobin (HbA1c), the patients were categorized into two groups: the TBP (n=43) and the TBDM (n=30) groups. The TBDM patients were characterized by an elevated *Mycobacterium tuberculosis*-specific QFT-P IFN-γ response after TB treatment compared to the TBP group (p<0.001 and p<0.05, respectively, after TB1 and TB2 antigens stimulation). A significantly higher proportion of positive QFT-P tests was observed in the TBDM group compared to the TBP group (91.3% vs 64.1%) at the end of the treatment (p=0.03). MLR analysis showed a decrease of MLR value after TB treatment for both diabetic and nondiabetic TB patients (p<0.001 and p<0.05). These data reflected from immune-host based tests used to monitor the TB treatment, seemed to further suggest that TB with concomitant DM is associated with a persistent inflammatory response after TB treatment.

## 1 Introduction

The World Health Organization (WHO) has identified diabetes mellitus (DM) as an important risk factor for tuberculosis (TB) and therefore recommends diabetes screening for active TB patients (1). Low- and middle-income countries account for approximately 80% of the global diabetes burden, and more than 90% of the global TB burden (2). For most patients, TB therapy provides a cure, but treatment failure and relapse can occur. It is now well established that DM is associated with an increased risk of progressing from latent TB infection to active TB disease. Moreover, TB patients with DM more frequently suffer from adverse TB outcomes, including delayed sputum conversion on treatment, TB treatment failure, death, and recurrent TB (1, 3–5). These outcomes are associated with moderate to severe adverse effects and long treatment durations that induce a lack of patient adherence to the treatment regimen and promote the emergence of drug resistance. Continuous monitoring and early identification of people with TB who are at risk of poor treatment outcomes could reduce the number of people who do not complete treatment. The WHO currently recommends sputum smear microscopy or mycobacteriological culture conversion at the end of the intensive phase of treatment for monitoring treatment response in adults with pulmonary TB (6). However, these microbiology-based methods rely on sputum samples, which are not readily available in all populations (e.g., pediatric TB, people living with HIV, extrapulmonary TB). Furthermore, while smear microscopy is related to poor sensitivity and specificity for outcome prediction, the TB culture has limited availability in primary care settings, and the delay in time to results constrains its clinical use.

There are novel tests and biomarkers in the pipeline that offer the potential to monitor TB treatment efficacy, predict outcomes, identify cure, and allow optimization of management. These potential tests include those using host characteristic assays, including assays for cytokines, transcriptomic profiles, and other biomarkers that are associated with the inflammatory reactions following TB infection. Some of these proposed tests, such as QuantiFERON-TB Gold Plus (QFT-P), white blood cell count (WBC) and the monocyte-to-lymphocyte ratio (MLR), are already commercialized to detect TB infection or used as routine basic laboratory measures for clinical practice. The QFT-P test, while primarily designed and validated for detecting *Mycobacterium tuberculosis* infection and guiding prophylactic treatment decisions in populations at higher risk of developing TB, has also been explored in research settings for its potential utility in detecting TB disease and monitoring TB treatment (7, 8). Patients with diabetes often have more severe inflammation at the time they are diagnosed with TB and experience higher risks for adverse TB treatment outcomes, with more severe lung damage in patients with pulmonary TB. This can have serious consequences like relapse or death, as frequently reported in TB patients with concomitant DM (TBDM) (4, 9). This inflammatory status can also alter the performance of host-immune-based assays’ capacity to monitor TB treatment (3, 10–12). The aim of this study was to assess the influence of DM on the respective performances of QFT-P and WBC, two available tests already used in clinical practice, in their capacities to monitor the TB treatment of drug-sensitive pulmonary TB. Considering the significant worldwide occurrence of both TB and DM, integrating changes related to DM in TB immunodiagnostic and immuno-monitoring tests can enhance the care provided to TB patients.

## 2 Materials and methods

### 2.1 Study design and participants

We conducted a prospective cohort study from January to December 2019 to consecutively recruit newly confirmed active TB patients from individuals with presumptive pulmonary TB seeking diagnosis at the main anti-TB center in Antananarivo, Madagascar. The inclusion criteria for active TB disease were adult pulmonary TB patients (≥ 18 years old) who tested positive for conventional TB microbiological and molecular tests, and able to provide informed consent. For each included TB participant, sociodemographic information was collected, including age, sex, body mass index (BMI, calculated as weight in kilograms divided by height in meters squared), BCG vaccination status, alcohol consumption and smoking habits. Two control groups (≥ 18 years old) were randomly and simultaneously recruited, including i) community healthy volunteers (HC) without any clinical signs/symptoms of TB, recruited at the anti-rabic center of the Institut Pasteur de Madagascar, and ii) confirmed DM patients without any clinical signs/symptoms of TB, recruited at the main Diabetes center of Antananarivo (AMADIA). Individuals who tested positive for HIV, children under 18 years of age, and individuals with other known comorbidities were excluded.

### 2.2 TB and DM diagnosis

Pulmonary TB is confirmed with sputum AFB smear microscopy using the auramine technique and/or the Loweinsten-Jensen (LJ) solid media culture and/or Xpert MTB/RIF at inclusion. The results were classified based on bacterial load as follows: AFB smear microscopy grades: 0 (no AFB observed), 1+ (10–99 AFB in 100 fields), 2+ (1–10 AFB per field in 50 fields), and 3+ (more than 10 AFB per field in at least 20 fields). Culture grades: 0 (no colonies), 1+ (1–100 colonies), 2+ (more than 100 discrete colonies), and 3+ (confluent growth or innumerable colonies).

DM was screened at the time of recruitment of the confirmed TB patients with an initial fasting plasma glucose (cut-off point at ≥ 6.1 mmol/L), and two points raised of glycated hemoglobin (HbA1c) were offered as confirmatory tests. Laboratory measurement of HbA1c with a diagnostic cut-off point ≥ 6.5% was used as the gold standard for the diagnosis of diabetes. To account for potential transient hyperglycemia, we made secondary analyses, defining diabetes by repeated raised HbA1c at the end of the TB treatment. HbA1c was measured in 1 mL of whole blood collected in ethylenediaminetetraacetic acid (EDTA) tubes and processed using immunoturbidimetry (Quest, Tucker, GA, USA). DM status was classified according to the American Diabetes Association guidelines (13) with a slight adjustment based on age-dependent HbA1c reference intervals (14).

### 2.3 TB treatment and follow-up visits

All patients were treated with the Directly Observed Treatment, Short Course (DOTS) and received the same TB treatment according to the WHO Drug-susceptible tuberculosis treatment recommendation (15) with a 6-month regimen composed of four first-line TB medicines: isoniazid (H, oral dose of 4-6 mg/kg/day), rifampicin (R, oral dose of 8-12 mg/kg/day), pyrazinamide (Z, oral dose of 20-30 mg/kg/day), and ethambutol (E, oral dose of 15-25 mg/kg/day). The regimen is a combination of those four drugs (HRZE) for 2 months followed by isoniazid and rifampicin (HR) for 4 months, administered daily. TB patients were followed up at the end of therapy after 6 months (M6) and at two months after the end of therapy (M8). At each of the three visits, a sputum sample and blood were collected for AFB smear microscopy, LJ culture, QFT-P assay, and WBC count.

### 2.4 QuantiFERON-TB gold plus assay

To perform the QFT-P and WBC count, 7 mL of whole blood was drawn in lithium heparin blood collection tubes. Four (4) mL were used for the QFT-P assay, and the remaining 3 mL were used for complete WBC, HbA1c, and the HIV test. The QFT-P assay was performed according to the manufacturer’s instructions (Qiagen). Briefly, venous blood was collected in lithium-heparin tubes at the health centers, and then one mL of blood was dispensed into each of the four QFT-P assay tubes (Nil, TB1, TB2, Mitogen) where antigen stimulations were initiated within 8 hours from venipuncture and incubated at 37˚C for 16 ± 24 hours (aiming at 18 hours’ incubation time). After incubation, the tubes were centrifuged, and aliquots of the plasma supernatants were stored at -20˚ C. IFN-γ ELISA was performed in batches according to the QFT-P protocol (Qiagen). ELISA results were converted to international units per milliliter (IU/mL) and interpreted using the QFT-P software supplied by the manufacturer (TB Gold Plus Analysis Software v2.71). All IFN-γ concentrations were nil-corrected. The results were classified as positive, negative, or indeterminate according to the manufacturer’s instructions, with a diagnostic IFN-γ cut-off of 0.35 IU/mL in either of the two antigen tubes. QFT-P conversion was defined as a change from negative to positive, and reversion as a change from positive to negative on serial testing.

### 2.5 White blood cells count assay

Complete WBC was performed with an XN 1000 automated hematology analyzer (Sysmex). The XN-1000 is a standalone, benchtop analyzer using a single XN-Series module. The XN-1000 automated analyzer provides a complete WBC and nucleated red blood cell (NRBC) count using the White Count and Nucleated Red Blood Cells (WNR) and the White blood cell differential count (WDF) channels. The blood cells are analyzed by flow cytometry-based optical measurement after red cell and platelet lysis, membrane permeabilization of the leukocytes, and introduction of a fluorochrome that binds to leukocyte nucleic acids. Scattergrams are generated after three-dimensional analysis of each cell signal according to cell volume (FSC: forward scatter light), cell structure (SSC: side scatter light), and cell fluorescence (SFL: side fluorescent light). The WNR channel evaluates the leukocyte and basophil counts and provides a systematic NRBC count. The WDF channel provides a count of the neutrophils, lymphocytes, eosinophils, monocytes, immature granulocytes, and a high-fluorescence lymphocyte count (HFLC). The monocyte-to-lymphocyte ratio (MLR) was determined by dividing absolute monocyte counts by absolute lymphocyte counts at each study time point.

### 2.6 Data collection and statistical analysis

Sociodemographic, clinical, and biological data were recorded in a dedicated RedCap^®^ (Research Electronic Data Capture) software database (16). Statistical analysis was performed using GraphPad Prism (version 9 for Windows, GraphPad Software, Boston, Massachusetts USA). Categorical variables were analyzed using Fisher’s exact test adjusted with Bonferroni’s *post-hoc* test (17). Normal continuous variables were analyzed with Student’s t-test and One Way ANOVA test. Non-normal continuous variables were analyzed with Mann-Whitney and Wilcoxon sum-rank tests for impaired and paired analysis respectively, and by One Way ANOVA test with Dunn’s Kruskal-Wallis rank sum-test (impaired) and Friedmann test (paired) for multiple comparisons *post-hoc* test (18).

### 2.7 Ethical considerations

This study was approved by the Ethical Committee for Biomedical Research of Madagascar (N° 099/MSANP/SG/AGMED/CERBM). Written informed consent was obtained prior to enrolment. All research was performed in accordance with relevant guidelines/regulations.

## 3 Results

### 3.1 Enrolment, clinical characteristics, follow-up visits, and sociodemographic data of study participants

**Figure 1:**
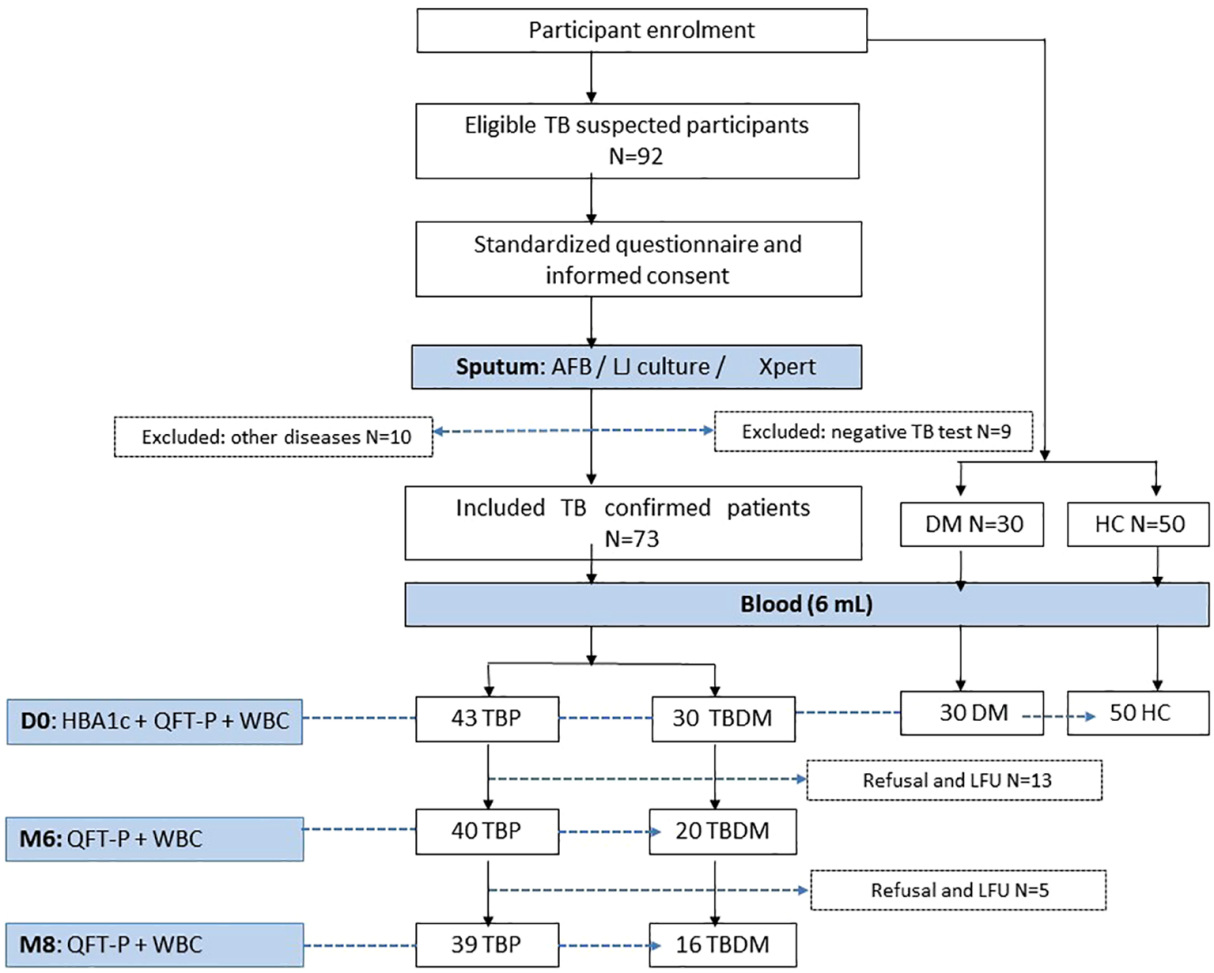
Flowchart of inclusion, follow-up visits and data collection process. AFB, acid fast bacilli; TBP, tuberculosis patient without diabetes mellitus; TBDM, tuberculosis patient with diabetes mellitus; DM, patient with diabetes mellitus; HC, healthy asymptomatic individuals; QFT-P, QuantiFERON-TB Gold Plus; WBC, White blood cell count; LFU, lost to follow up; HBA1c, glycated hemoglobin A1C; LJ, Löwenstein Jensen; Xpert, Xpert MTB/RIF Ultra.

**Figure 1** shows the enrolment and follow-up visit flowchart of the study participants. Among the 92 eligible participants in the TB active group, a total of 73 newly confirmed pulmonary TB patients were included in the study. A total of 10 participants were excluded due to the following health conditions: high blood pressure (n=5), asthma (n=2), meningitis (n=1), measles (n=1), and gastritis (n=1). Additionally, 9 patients were excluded for having a negative TB test. Most patients were simultaneously positive for sputum smear microscopy (82%, 60/73) and/or culture (89%, 65/73) and/or Xpert (100%, 73/73). After laboratory measurement of HbA1c, these patients were categorized into 2 groups: the active pulmonary group without DM (TBP, n=43) and the active pulmonary with DM groups (TBDM, n=30) (**Figure 1**). Control groups including Diabetes only (DM, n=30) and healthy asymptomatic blood donors (HC, n=50) were enrolled. The clinical groups with DM (TBDM and DM) had a statistically significant higher age compared to HC groups (p<0.0001, **Table 1**). However, there was no statistical age difference between TBDM and TBP patients (p=0.058). A statistically significant lower body mass index (BMI) was observed with the TBP (17.6 Kg/m2) and TBDM (18.4 Kg/m2) groups compared to the DM group (23.1 Kg/m2) (p<0.0001). The frequency of underweight individuals (BMI<18.5) was significantly higher in both the TBP and TBDM groups compared to the DM group (p<0.0001), highlighting the pronounced impact of TB on nutritional status in affected individuals. **Table 1** also shows that a statistically significant higher alcohol consumption (p<0.003) and higher smoker proportions (p=0.008) were observed within the TBP compared to TBDM. Then, in the downstream analysis, we decided to only perform with TB patients (TBP and TBDM) who simultaneously do not smoke and do not drink alcohol. Regarding the follow-up visits, 39 TBP and 16 TBDM patients had successfully achieved their TB treatment and completed their QFT-P and WBC count assays from the inclusion (D0), at M6 to the M8 follow-up visits (**Figure 1**). Respectively, 40 and 20 patients were followed until the end of the TB treatment for TBP and TBDM (**Figure 1**). All of them presented negative TB culture at the end of their treatment and were clear from any clinical TB signs at M6. Among the TBDM patient, only five individuals (n=5) did receive anti-diabetic treatment in parallel with their anti-TB treatment. **Table 2** shows the degree of positivity of AFB smear microscopy and culture results within the TBP and TBDM groups at inclusion (D0). No significant difference was observed between the TBP and TBDM patients regarding the degree of positivity of AFB smear, culture and Xpert results.

**Table 1:** Sociodemographic and clinical characteristics of study participant at inclusion.

Study demographics	TBP	TBDM	DM	HC	p-value TB vs TBDM	p-value All group
**Age** (Median, range)	31 (22-45)	43 (35-56)	59 (38-63)	34 (22-44)	0.058	<0.0001
Sex (N, %)						
Male	32 (74.4)	19 (63.3)	9 (30.0)	15 (30.0)	0.43	<0.0001
Female	11 (25.6)	11 (36.7)	21 (70.0)	35 (70.0)		
**BMI** (Kg/m^2^) (Median, range)	17.2 (16.0-18.9)	18.0 (16.6-19.2)	22.9 (21.2-25.0)		0.18	<0.0001
BMI category						
Underweight	34 (79.0)	18 (60.0)	1 (3.3)		0.20	<0.0001
Normal weight	8 (18.7)	11 (36.7)	21 (70.0)			
Overweight	1 (2.3)	1 (3.3)	8 (26.7)			
BCG Vaccination (N, %)						
Yes	32 (74.4)	27 (90.0)	29 (96.7)	46 (92.0)	0.10	0.01
No	11 (25.6)	3 (10.0)	1 (3.3)	4 (8.0)		
**HbA1c %** (Median, range)	5.6 (5.4-5.9)	8.2 (7.0-9.1)	9.3 (7.6-11.1)	4.5 (3.4-5.1)	<0.0001	<0.0001
Alcohol (N, %)						
Yes	24 (55.8)	6 (20.0)	3 (10.0)		0.003	<0.0001
No	19 (44.2)	24 (80.0)	27 (90.0)			
Smoking (N, %)						
Yes	24 (55.8)	7 (23.3)	4 (13.3)		0.008	0.0003
No	19 (44.2)	23 (76.7)	26 (86.7)			
**Total**	43	30	30	50		

TBP, tuberculosis patient without diabetes mellitus; TBDM, tuberculosis patient with diabetes mellitus; DM, patient with diabetes mellitus; HC, healthy asymptomatic individuals; BMI, Body mass index; BCG, Bacille Calmette and Guerin; HBA1c, Glycated hemoglobin A1C; N, number.

**Table 2:** Comparison of AFB smear, culture and Xpert results between TBP and TBDM groups at inclusion (D0).

	Grade	TBP (n, %)	TBDM (n, %)	P-value
**AFB Smear**	0 1+ 2+ 3+	8 (18.6) 13 (30.2) 12 (27.9) 10 (23.3)	8 (26.7) 9 (30.0) 8 (26.7) 5 (16.6)	0.82
**Culture**	0 1+ 2+ 3+	5 (11.6) 10 (23.2) 13 (30.2) 15 (35.0)	6 (20.0) 5 (16.7) 13 (43.3) 6 (20.0)	0.9
**Xpert**	Not detected Low Medium High	2 (4.6) 5 (11.6) 9 (21.0) 27 (62.8)	3 (10.0) 6 (20.0) 6 (20.0) 15 (50.0)	0.55
**Total**		43	30	

TBP, tuberculosis patient without diabetes mellitus; TBDM, tuberculosis patient with diabetes mellitus; AFB, acid-fast bacilli.

### 3.2 QuantiFERON-TB gold plus results

In order to evaluate the impact of DM on IFN-γ production upon *Mycobacterium tuberculosis* antigen stimulation during TB treatment, the plasma IFN-γ concentrations in response to TB1 and TB2 antigens were first measured with the QFT-P assay and compared between the TBP, TBDM, DM, and HC clinical groups. At inclusion (D0), no statistical difference in the proportion of QFT-P positive, negative, and indeterminate results was observed between the TBP vs TBDM nor between the other study groups (p=0.2) (**Table 3**). Furthermore, after quantitative analysis, no statistical differences in the baseline level of IFN-γ produced in QFT-P positive results after stimulation with TB1 (p=0.11) and TB2 (p=0.19) antigens were observed between the clinical groups (**Supplementary Figure 1**). Additionally, our results did not reveal any statistically significant differences in IFN-γ levels among the various smear and culture grades within the TBP and TBDM groups (p>0.05). The results revealed weak correlations between IFN-γ levels and both smear/culture grades and Xpert-derived bacillary load.

**Table 3:** QuantiFERON-TB Gold Plus results with the 4 study groups at inclusion.

QFT-P	TBP (n, %)	TBDM (n, %)	DM (n, %)	HC (n, %)	P-value
**POS**	25 (58.1)	17 (56.7)	14 (46.7)	25 (50.0)	0.2
**NEG**	15 (34.9)	10 (33.3)	16 (53.3)	23 (46.0)	
**IND**	3 (7.0)	3 (10.0)	0 (0.0)	2 (4.0)	
**Total**	43	30	30	50	

TBP, tuberculosis patient without diabetes mellitus; TBDM, tuberculosis patient with diabetes mellitus; DM, patient with diabetes mellitus; HC, healthy asymptomatic individuals; QFT-P, QuantiFERON-TB Gold Plus; POS, positive; NEG, negative; IND, indeterminate.

**Figure 2:**
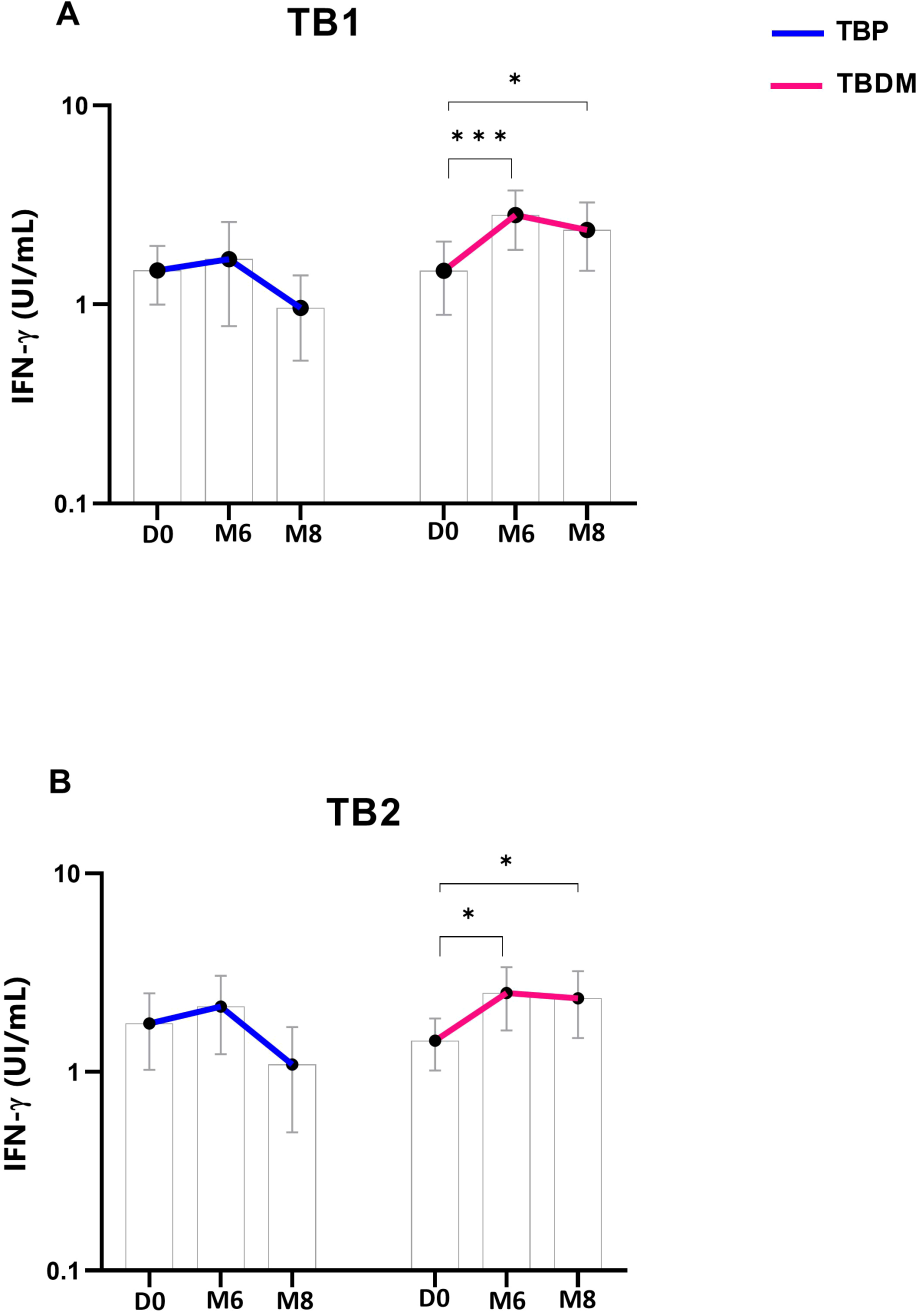
Dynamics of QuantiFERON-TB Gold Plus interferon-γ concentrations in TBP and TBDM groups throughout TB treatment: **(A)** Stimulation by TB1-specific antigen and **(B)** by TB2-specific antigen. Data represent median with 95% confidence interval. Data were analyzed using Friedman’s multiple pairwise comparisons test. *: p < 0.05, ***: p < 0.001. TBP, tuberculosis patient without diabetes mellitus; TBDM, tuberculosis patient with diabetes mellitus; QFT-P, QuantiFERON-TB Gold Plus; D0, baseline before treatment initiation; M6, end of treatment, after six month; M8, 2 months after the end of treatment.

**Figure 3:**
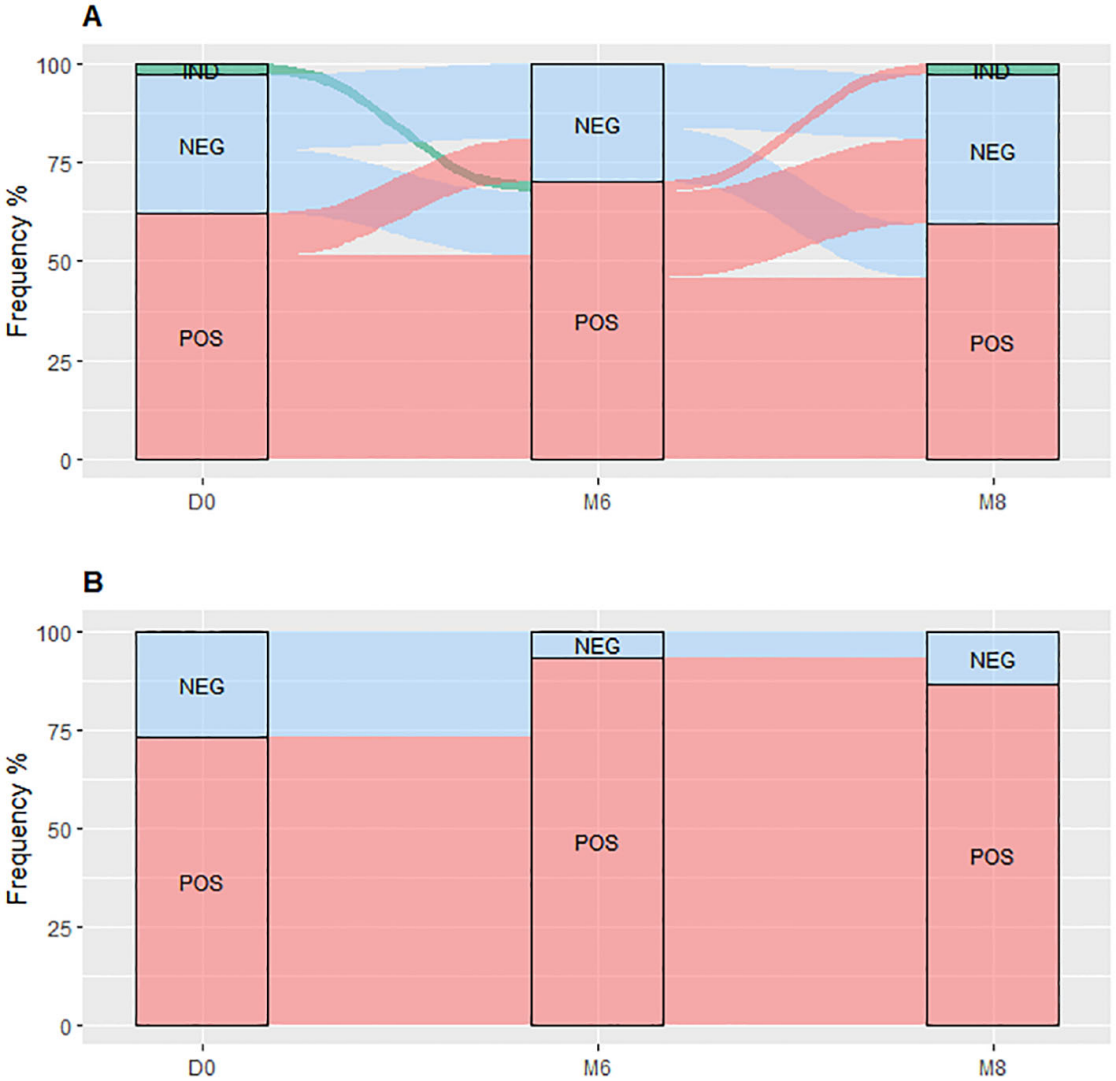
QuantiFERON-TB Gold Plus results dynamic proportion with the TBP **(A)** and TBDM **(B)** throughout TB treatment. TBP, tuberculosis patient without diabetes mellitus; TBDM, tuberculosis patient with diabetes mellitus; QFT-P, QuantiFERON-TB Gold Plus; POS, positive; NEG, negative; IND, indeterminate; D0, baseline before treatment initiation; M6, end of treatment, after six month; M8, 2 months after the end of treatment.

TBP and TBDM had a follow-up visit at M6 from their TB treatment and two months after the completion of the TB treatment (M8) timepoints. TBDM group showed higher IFN-γ produced by the QFT-P after stimulation with both antigens, TB1 (p<0.001) and TB2 (p<0.05), at M6 compared to D0. In contrast no significant differences were observed in the TBP group at these timepoints or between M6 and M8 (**Figure 2**). When applying the manufacturer’s recommendations for qualitative results, while no statistical difference of QFT-P results was observed between the two clinical groups at inclusion (D0) and M8, a significantly higher proportion of positive QFT-P results was observed for the TBDM group compared to the TBP group (91.3% vs 64.1%) (p=0.03) at the achievement point of the treatment (M6) (**Figure 3**). During follow up, there were no significant differences in QFT-P responses between TBDM patients receiving anti-diabetic treatment and those not receiving it (p>0.05).

### 3.3 QuantiFERON-TB gold plus conversion/reversion rates

The QFT-P conversion and reversion rates during TB treatment showed that the TBDM patients had higher conversion rates (83.3% for TBDM vs 38.4% for TBP, p<0.05) and no reversions (0% for TBDM vs 21.7% for TBP, p<0.01) compared to the TBP at the end of the TB treatments (M6). Notably, five out of six TBDM patients with a negative QFT-P result at D0 had QFT-P conversion at M6, and 1/6 remained QFT-P negative at M6 (**Figure 3B**). Besides, none of the TBDM patients who had a positive QFT-P result at D0 reverted their QFT-P test as negative, and all remained QFT-P positive at M6 (**Figure 3B**). No statistical differences in QFT-P indeterminate proportion between the groups at the different follow-up visits were observed (**Figure 3**).

### 3.4 WBC, monocyte and lymphocyte counts

**Figure 4:**
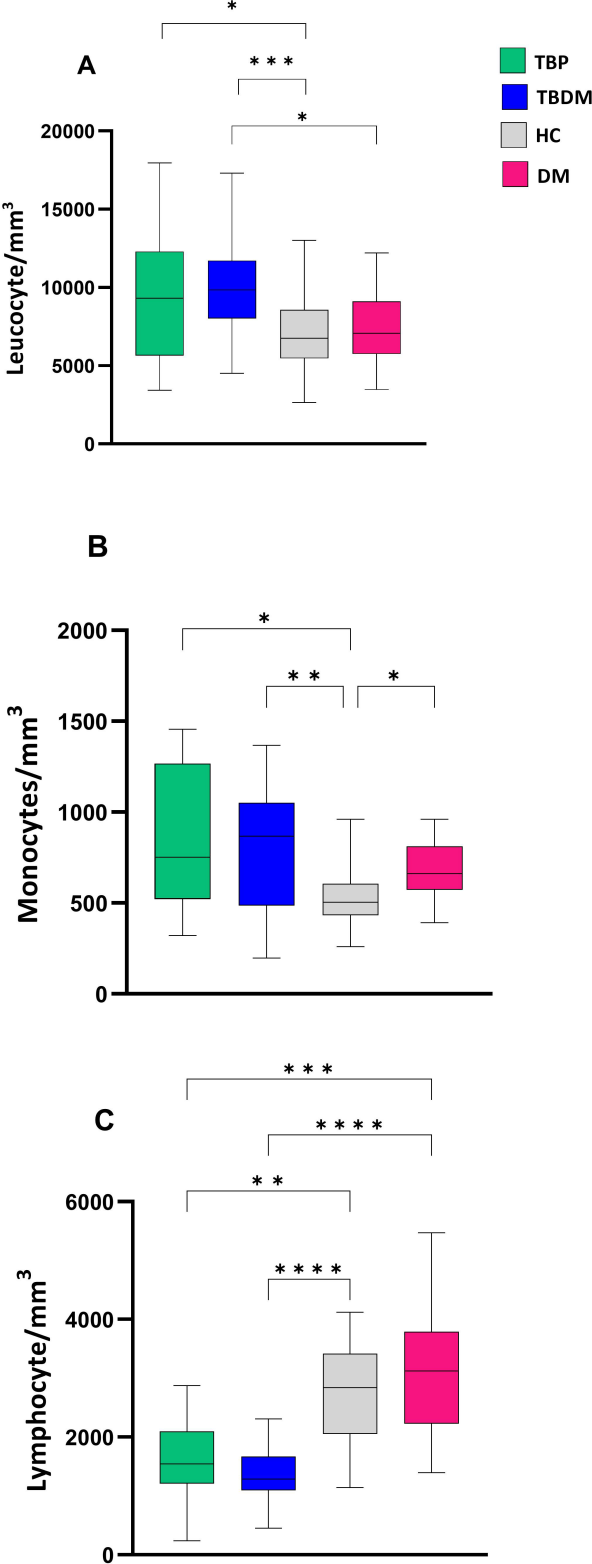
Comparison of baseline absolute white blood cell count between TBP, TBDM, HC and DM. **(A)** Leucocyte absolute count/mm^3^ of whole blood. **(B)** Monocyte absolute count/mm^3^ of whole blood. **(C)** Lymphocyte absolute count/mm^3^ of whole blood. Box plots represent median and interquartile range (IQR) of the data. The upper and lower edges of the boxes represent the third and first quartiles (Q3 and Q1), respectively, while the line inside the box represents the median (Q2). Data were analyzed using Kruskal-Wallis with Dunn’s multiple comparison test. *: p < 0.05, **: p < 0.01, ***: p < 0.001, ****: p < 0.0001. TBP, tuberculosis patient without diabetes mellitus; TBDM, tuberculosis patient with diabetes mellitus; DM, patient with diabetes mellitus; HC, healthy asymptomatic individuals.

Before the initiation of TB treatment, the TB active groups (TBP and TBDM) had higher absolute WBC counts compared to the healthy control group (HC) (p<0.05 and p<0.001) (**Figure 4A**). TBDM also showed a significantly higher WBC count compared to its DM clinical counterpart group (p<0.05) (**Figure 4A**). **Figures 4B, C** show that TBDM and TBP patients displayed statistically significant higher monocyte (p<0.01 and p<0.05) with lower lymphocyte counts compared to HC and DM groups. The results did not reveal any significant associations between bacterial burden (by AFB smear, culture and Xpert) and leukocyte counts at inclusion within the two groups.

**Figure 5:**
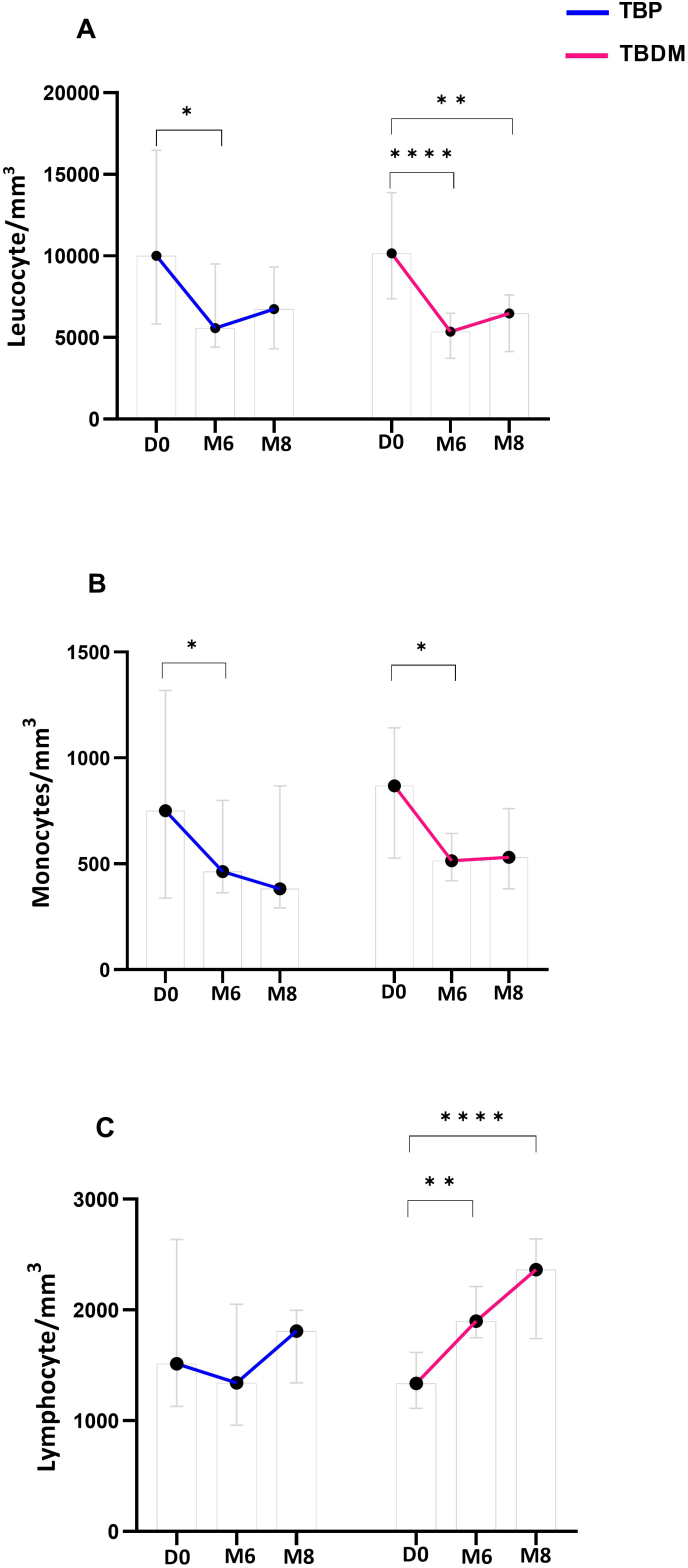
Dynamics of main white blood cell types proportion throughout TB treatment in TBP and TBDM groups. **(A)** Leucocyte absolute count/mm3 of whole blood. **(B)** Monocyte absolute count/mm3 of whole blood. **(C)** Lymphocyte absolute count/mm3 of whole blood. Data represent median with 95% confidence interval. Data were analyzed using Friedman’s multiple pairwise comparisons test. *: p < 0.05, **: p < 0.01, ****: p < 0.0001. TBP, tuberculosis patient without diabetes mellitus; TBDM, tuberculosis patient with diabetes mellitus; D0, baseline before treatment initiation; M6, end of treatment, after six month; M8, 2 months after the end of treatment.

After their TB treatment, significant decreases in absolute WBC counts were observed in the TBP and TBDM clinical groups at both M6 and M8 (**Figure 5A**). Moreover, a significant decrease in monocyte absolute count within the TBP and TBDM group was observed after treatment compared to inclusion (p<0.05) (**Figure 5B**). While a statistically significant increase in lymphocyte absolute count was observed in the TBDM group after treatment both at M6 (p<0.01) and M8 (p<0.0001), no significant increase in lymphocyte absolute count was observed with the TBP after TB treatment (**Figure 5C**).

### 3.5 Monocyte-to-lymphocyte ratio analysis

**Figure 6:**
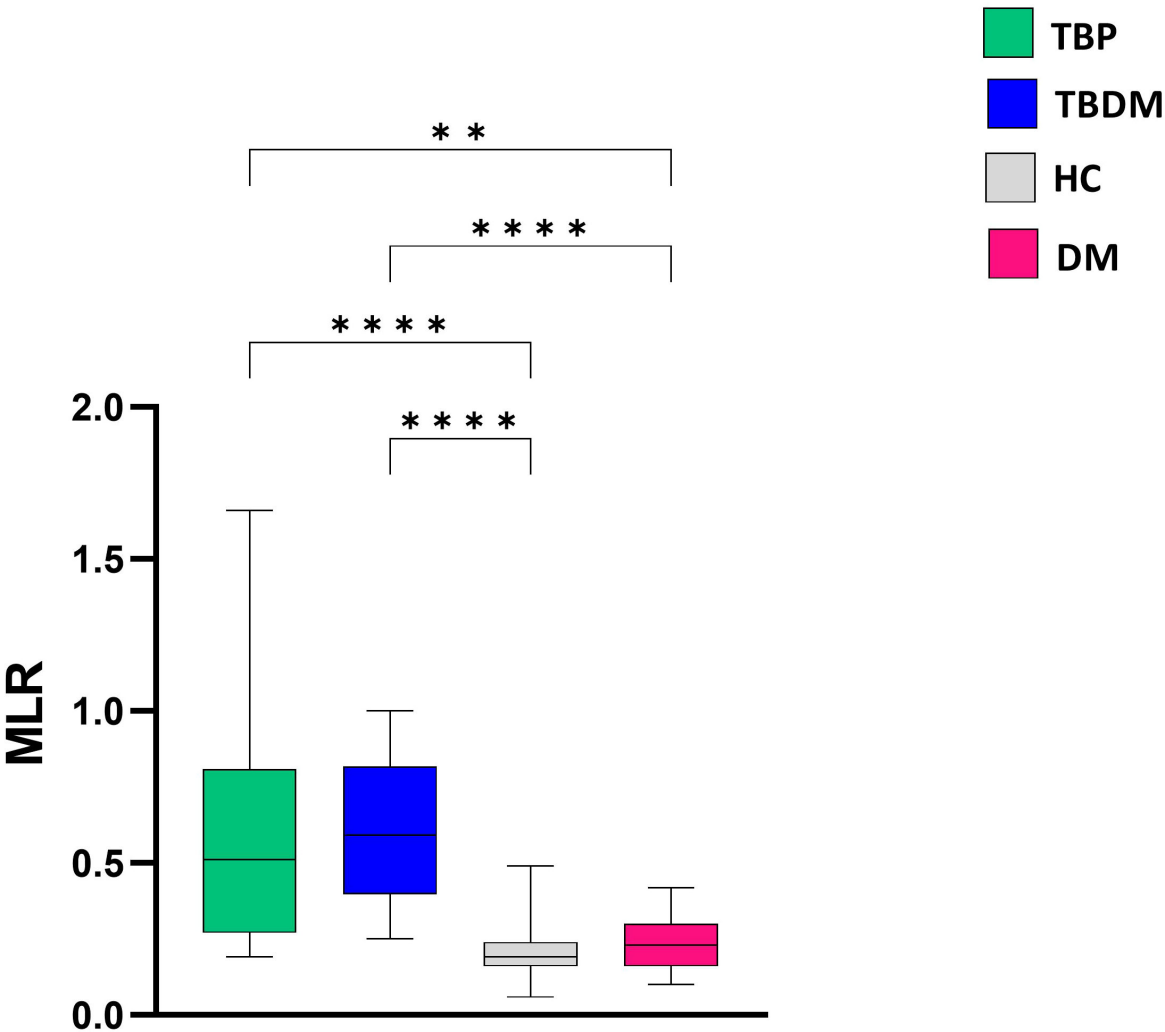
Comparison of baseline monocyte-to-lymphocyte (MLR) ratio between TBP, TBDM, HC and DM groups. Box plots represent median and interquartile range. The upper and lower edges of the boxes represent the third and first quartiles (Q3 and Q1), respectively, while the line inside the box represents the median (Q2). Data were analyzed using Kruskal-Wallis with Dunn’s multiple comparison test. **: p<0.01, ****: p < 0.0001. TBP, tuberculosis patient without diabetes mellitus; TBDM, tuberculosis patient with diabetes mellitus; DM, patient with diabetes mellitus; HC, healthy asymptomatic individuals.

**Figure 7:**
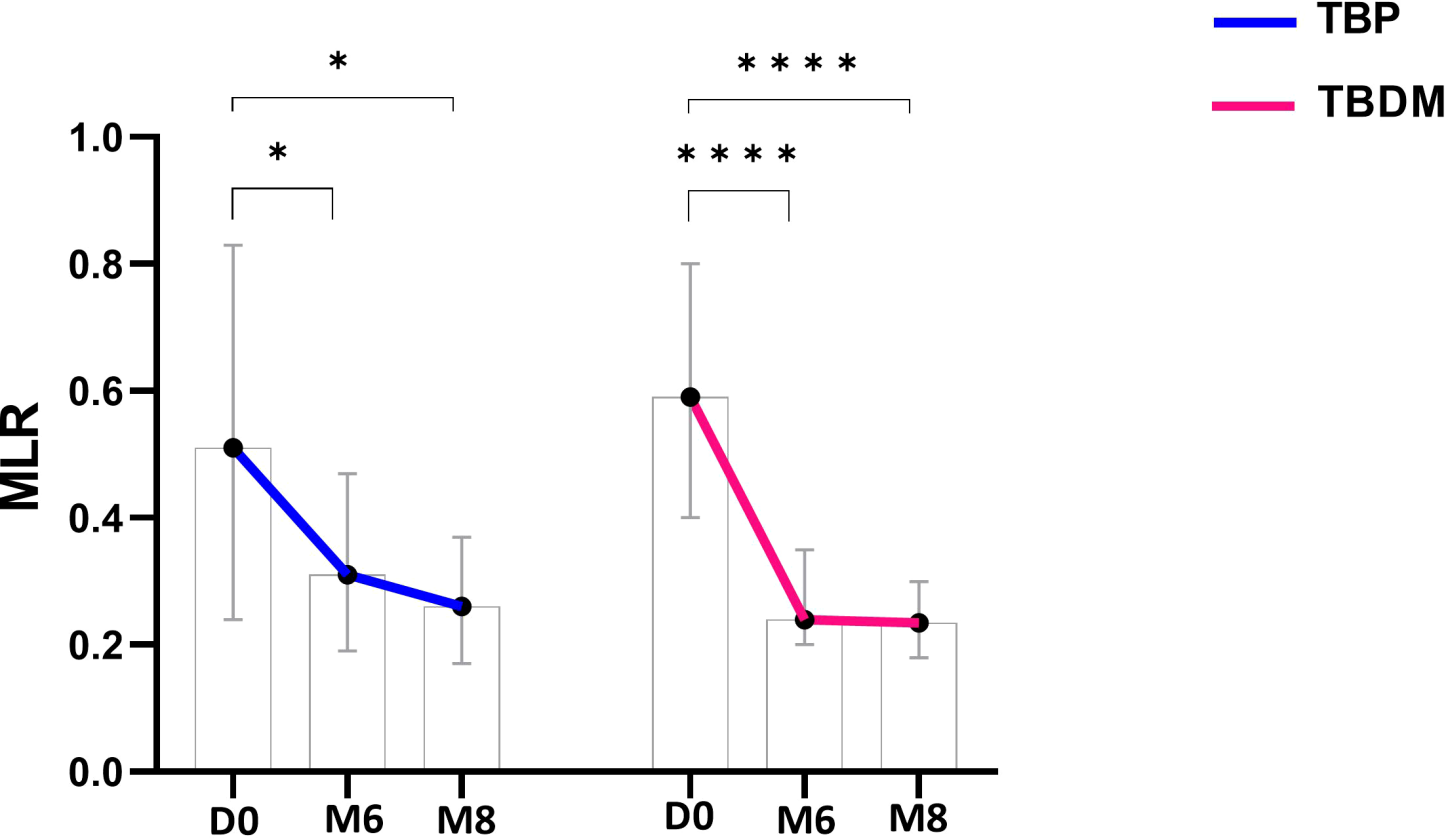
Dynamics of monocyte-to-lymphocyte (MLR) ratio throughout TB treatment with TBP (blue line) and TBDM (red line) groups. Data represent median with 95% confidence interval. Data were analyzed using Friedman’s multiple pairwise comparisons test. *: p < 0.05, ****: p < 0.0001. TBP, tuberculosis patient without diabetes mellitus; TBDM, tuberculosis patient with diabetes mellitus; D0, baseline before treatment initiation; M6, end of TB treatment, after six month; M8, 2 months after the end of TB treatment.

After determining the MLR at inclusion, a significantly higher ratio was observed for both TBP and TBDM groups compared to their respective control counterparts, i.e., HC (p<0.0001) and DM groups (p<0.01 and p<0.0001, respectively for TBP and TBDM) (**Figure 6**). After the TB treatment, a significant decrease in the MLR was observed both at M6 and M8 in the TBP group (p<0.05, **Figure 7**). The same trend was observed with the TBDM at M6 and M8 but with a higher statistical difference compared to the trend observed with the TBP (p<0.001). A significant decline of the MLR level was observed with both TB active group after successful TB treatment. No statistical differences were observed regarding the MLR at M6 and M8 compared to HC (**Supplementary Figure 2**). The same trends were observed when analyzing the neutrophil-to-lymphocyte ratio (NLR) in both groups (**Supplementary Figure 3**). At baseline (D0), significantly higher NLR values were observed, which steadily declined following successful TB treatment at M6 and M8. During follow up, there were no significant differences in MLR and NLR responses between TBDM patients receiving anti-diabetic treatment and those not receiving it (p>0.05).

## 4 Discussion

Host characteristic assays, which are based on monitoring the host immune system, are alternative sputum-independent options for monitoring and predicting TB treatment outcomes. These assays can be performed in primary health care settings, where TB patients often first enter the health care system (6). Most of the technologies used for monitoring TB treatment are still in the early stages of development and are limited to central laboratories. These available TB monitoring tests have also faced challenges due to the host’s heterogeneous variations, including co-morbidities associated with infection, as well as the practicalities of translating biomarkers into simple and rapid tests that are more suitable for low- and middle-income countries (LMICs) (8).

The aim of this study was to assess the influence of diabetes mellitus (DM) on the respective performances of QuantiFERON-TB Gold Plus (QFT-P) and white blood cell counts (WBC), two available tests already used in clinical practice, in their capacities to monitor the TB treatment of drug-sensitive pulmonary TB. While IGRA from QFT-P has already been reported to vary depending on the TB treatment course (7, 19), the influence of DM on the QFT-P was mainly reported in cases of TB infection detections. Our study is consistent with previous studies that identified persistent inflammation with notably systemic type 1 and pro-inflammatory response during anti-TB treatment in TBDM patients (9, 20–22). In the present study, DM appeared to not influence the QFT-P qualitative result proportions at baseline, with identical performance in TBP, TBDM, and DM clinical groups, and no significant difference was observed in the quantitative responses to TB1 and TB2 antigens between diabetics and nondiabetic TB patients at inclusion. However, higher *Mycobacterium tuberculosis*-specific responses were reported in infected patients with prediabetes and may reflect pathological inflammatory and ineffectual responses to TB or active *Mycobacterium tuberculosis* replication in participants with diabetes (23). After TB treatment, a systematic review of about 30 studies found that quantitative IGRA responses generally decrease during treatment for TB (19). However, in the present study, after TB treatment, a significantly higher IFN-γ response was observed in the TBDM patients compared to those without DM (TBP). Moreover, a higher conversion rate (83.3%) and a low reversion rate (0.0%) were observed within the TBDM patients, as all treated TBDM patients with positive QFT-P at baseline remained positive until the end of treatment at M6. This observed heightened IFN-γ response in TBDM compared to TBP raises intriguing questions regarding the interplay between these two conditions and their impact on immune responses to TB treatment. This finding aligns with previous studies indicating that DM can exacerbate inflammatory responses and alter immune function, potentially influencing treatment outcomes in TB (22, 24). We suggest that following successful TB treatment, improved glycemic control may help restore immune function, enabling T cells to regain their capacity to produce IFN-γ. In other hand, this improved glycemic control following successful TB treatment might not only restore immune function but also help modulate the high concentrations of IFN-γ that persist post-treatment. This regulatory effect could play a crucial role in re-establishing immune homeostasis. While the interferon-gamma overactivation or dysregulation is known to play a role in the development or exacerbation of dysglycemia (25) by for instance promoting insulin resistance and contribute to dysglycemia. Hyperglycemia on the other hand can indeed impair the function of immune cells which can result in chronic inflammation and tissue damage (26). Hyperglycemia and inflammation create a feedback loop in which high blood glucose levels promote the release of pro-inflammatory cytokines and other inflammatory mediators like the interferon-gamma. These, in turn, contribute to insulin resistance and chronic inflammation, worsening dysglycemia. Over time, this feedback loop can lead to serious complications, especially in conditions like type 2 diabetes, where inflammation and insulin resistance perpetuate one another. Managing blood glucose levels and controlling inflammation are crucial to breaking this cycle and preventing the progression of metabolic disorders. Additionally, we anticipate that pulmonary impairment post-TB could be more pronounced in patients with concurrent diabetes due to the prolonged inflammatory state. Our study may shed light on the heterogenous patterns of IGRA levels following treatment due to the high degree of variation between participants, which need to be adapted for DM and patients with similar dysregulated immune systems in order to be useful for monitoring anti-tuberculous treatment in clinical practice (27). The higher conversion rate and minimal reversion rate observed exclusively within the TBDM group suggest a distinct immune profile in these patients after treatment. The persistent positivity of QFT-P among all initially positive TBDM patients may simply imply a sustained immune activation despite treatment completion, which could reflect ongoing inflammation or impaired immune regulation in the context of DM (20–22).

Besides the QFT-P assay, we also performed WBC count analysis and monitored the monocyte to lymphocyte ratio (MLR) in the two groups (TBP and TBDM) prior to, during, and after completion of TB treatment. Prior to TB treatment, elevated WBC and monocyte counts with lymphopenia were observed in both groups of TB patients (TBP and TBDM). After TB treatment, WBC and monocyte absolute counts globally decreased for the two groups of TB patients treated (**Figure 5A**), indicating a systemic response to TB therapy, regardless of DM comorbidity. These findings suggest that while DM may predispose individuals to altered immune function and inflammatory responses (24), it does not significantly impact the overall dynamics of WBC levels in TB patients prior to treatment initiation. MLR has been observed to be associated with active TB and other studies have reported decreases in the MLR values after anti-TB treatment (28–31). Thus, the MLR assay can be used as a biomarker to identify TB and monitor the effectiveness of anti-TB therapy (30, 32). It has been reported that DM can modulate the dynamics of the immune cells, notably the monocytes and lymphocytes (33–36). In the TB field, DM could affect the basal activation state of some effector cells and their capacity to control *Mycobacterium tuberculosis* infection (37). However, in the present study, our results showed that DM does not globally affect the WBC dynamics in TB patients prior to treatment, and a MLR decrease was observed in both the TBP and TBDM groups after TB treatment. Thus, this reduction in MLR values following TB treatment in both groups reflects the efficacy of anti-tuberculosis therapy in modulating systemic inflammation, suggesting a restoration of immune balance and resolution of the inflammatory milieu associated with active TB infection (22). While further investigation should be conducted to assess the physical lung tissue damages and the persisting inflammation due to DM following TB treatment, it is worthwhile to notice that in the present study, like the TBP group, all the TBDM patients had successful TB treatment.

In this study, we did not show any differences in QFT-P or MLR results between TBDM patients receiving diabetes treatment and those not receiving it during TB follow-up. It suggests that diabetes management alone may not significantly alter immune response markers in TB treatment. This aligns with studies indicating that while diabetes can modulate immune function, glycemic control might not lead to measurable changes in TB-specific immune markers, such as IFN-γ release or lymphocyte reactivity. Chronic hyperglycemia in diabetic patients can induce a state of immune dysregulation that persists despite glucose-lowering interventions, affecting both innate and adaptive immunity without necessarily resulting in immediate changes in TB-specific immune responses during treatment (38). Additionally, diabetes is known to impact both pro-inflammatory and anti-inflammatory cytokine pathways, creating a complex immune environment that diabetes treatment alone may not fully normalize during the course of TB therapy (39). In this study, prediabetic patients were included in the TBDM group to enable a broader analysis of the spectrum of diabetes-associated TB. However, the relatively small number of participants in the prediabetic subgroup may have limited the statistical power to detect subtle immunological differences within this group. Based on the two points of HbA1c during the study period, none of the TBP patients changed to a diabetic status (TBDM), and none of the TBDM patients presented with transient DM.

In conclusion, our study provides insights into the complex interaction between TB and DM and their implications for treatment monitoring and immune response dynamics. Despite limitations in sample size, the limited number of TBDM patient receiving anti-diabetic treatment, the absence of treatment failure cases, and the prospective nature of our study, which does not account for retrospective biases or pre-existing health variations among participants, our findings highlight the distinct immune profiles observed in TB patients with and without DM, particularly regarding the performance of host immune-based assays such as QFT-P and MLR in monitoring treatment response. Our results suggest that, while DM may not globally affect WBC dynamics in TB patients prior to treatment, it can influence immune responses to TB treatment. This is evidenced by the heightened IFN-γ response and persistent QFT-P positivity observed in TBDM patient post-treatment, which may potentially contribute to immune-associated pathologies and poor clinical damage control. These findings highlight the importance of considering DM as a potential modifier of TB treatment outcomes and highlight the need for tailored monitoring strategies in this vulnerable population. To further investigate these dynamics, future studies should consider analyzing additional cytokines using the plasma samples stored in our biobank. Finally, while further research is needed to validate these findings in larger, more diverse patient cohorts and to explore the long-term implications of DM on TB treatment outcomes, our study emphasizes the significance of implementing comprehensive monitoring strategies and personalized approaches to TB management, particularly in regions with a high burden of both TB and DM.

## Data Availability

The raw data supporting the conclusions of this article will be made available by the authors, without undue reservation.

## Supplementary material

Supplementary Figure 1 IFN-γ value comparison between participants with positive QuantiFERON-TB Gold Plus among TBP, TBDM, DM and HC groups following stimulation with TB1 and TB2 antigens at inclusion (D0). Box plots represent median and interquartile range. The upper and lower edges of the boxes represent the third and first quartiles (Q3 and Q1), respectively, while the line inside the box represents the median (Q2).

Supplementary Figure 2 Monocyte-to-lymphocyte ratio (MLR) levels of TBP and TBDM at M6 and M8 compared to healthy controls (HC). Box plots represent median and interquartile range. The upper and lower edges of the boxes represent the third and first quartiles (Q3 and Q1), respectively, while the line inside the box represents the median (Q2).

Supplementary Figure 3 Comparison of neutrophil-to-lymphocyte ratio (NLR) levels after TB treatment (M6 and M8) among the TBP and TBDM groups, and healthy controls (HC). Box plots represent median and interquartile range. The upper and lower edges of the boxes represent the third and first quartiles (Q3 and Q1), respectively, while the line inside the box represents the median (Q2). Data were analyzed using Kruskal-Wallis with Dunn’s multiple comparison test. ***: p<0.001, ****: p < 0.0001.
