# Supplementary figures and images for: Assessing the effects of diabetes mellitus on the monocyte-to-lymphocyte ratio and the QuantiFERON-TB gold plus assays for tuberculosis treatment monitoring: a prospective cohort study
Source: Front Immunol. 2025 Jan 17;15:1451046. doi: 10.3389/fimmu.2024.1451046 (PMC11782029; doi:10.3389/fimmu.2024.1451046)

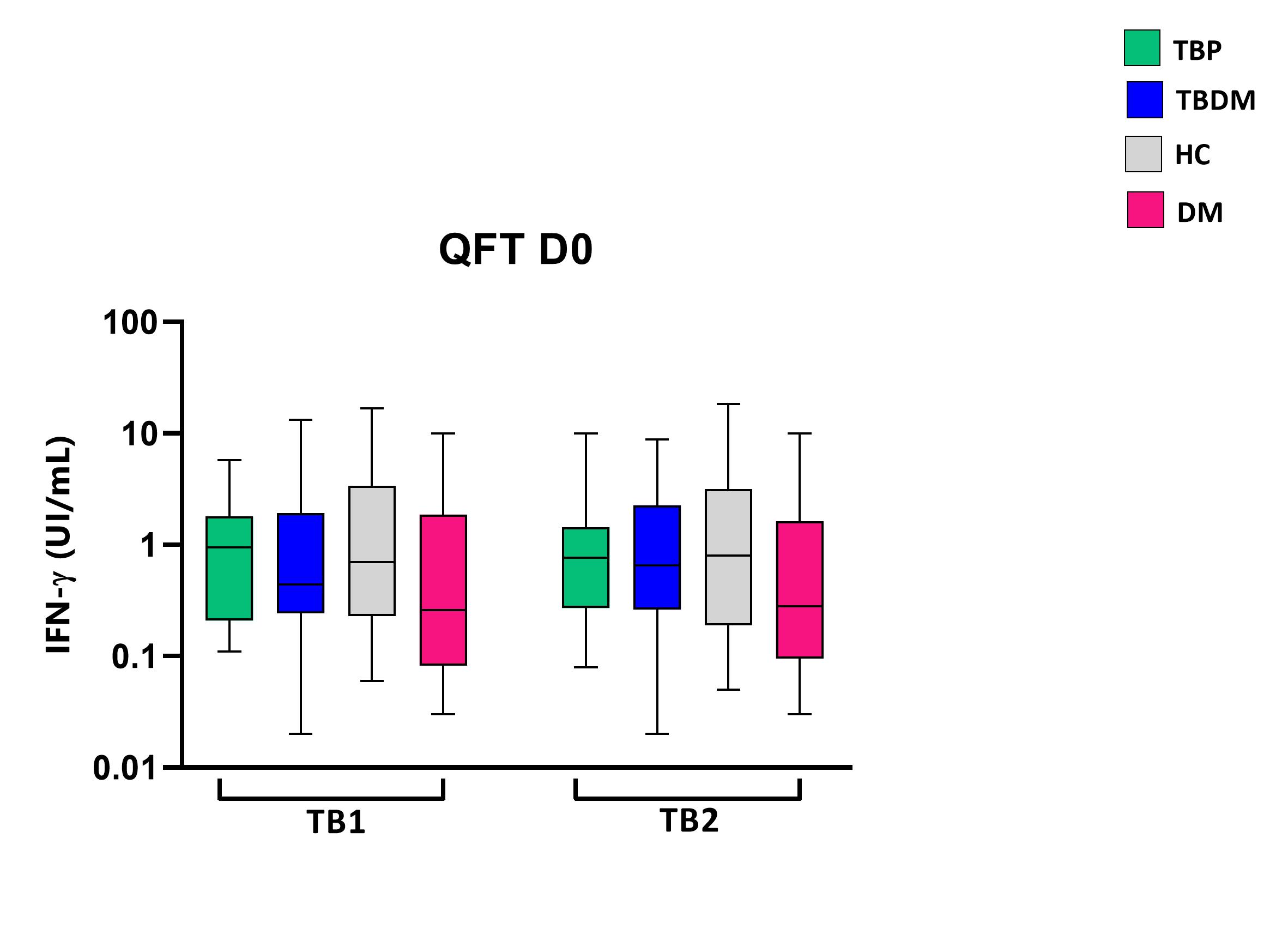

Supplement: Supplementary Figure 1 — IFN-γ value comparison between participants with positive QuantiFERON-TB Gold Plus among TBP, TBDM, DM and HC groups following stimulation with TB1 and TB2 antigens at inclusion (D0). Box plots represent median and interquartile range. The upper and lower edges of the boxes represent the third and first quartiles (Q3 and Q1), respectively, while the line inside the box represents the median (Q2). [file Image1.jpeg]

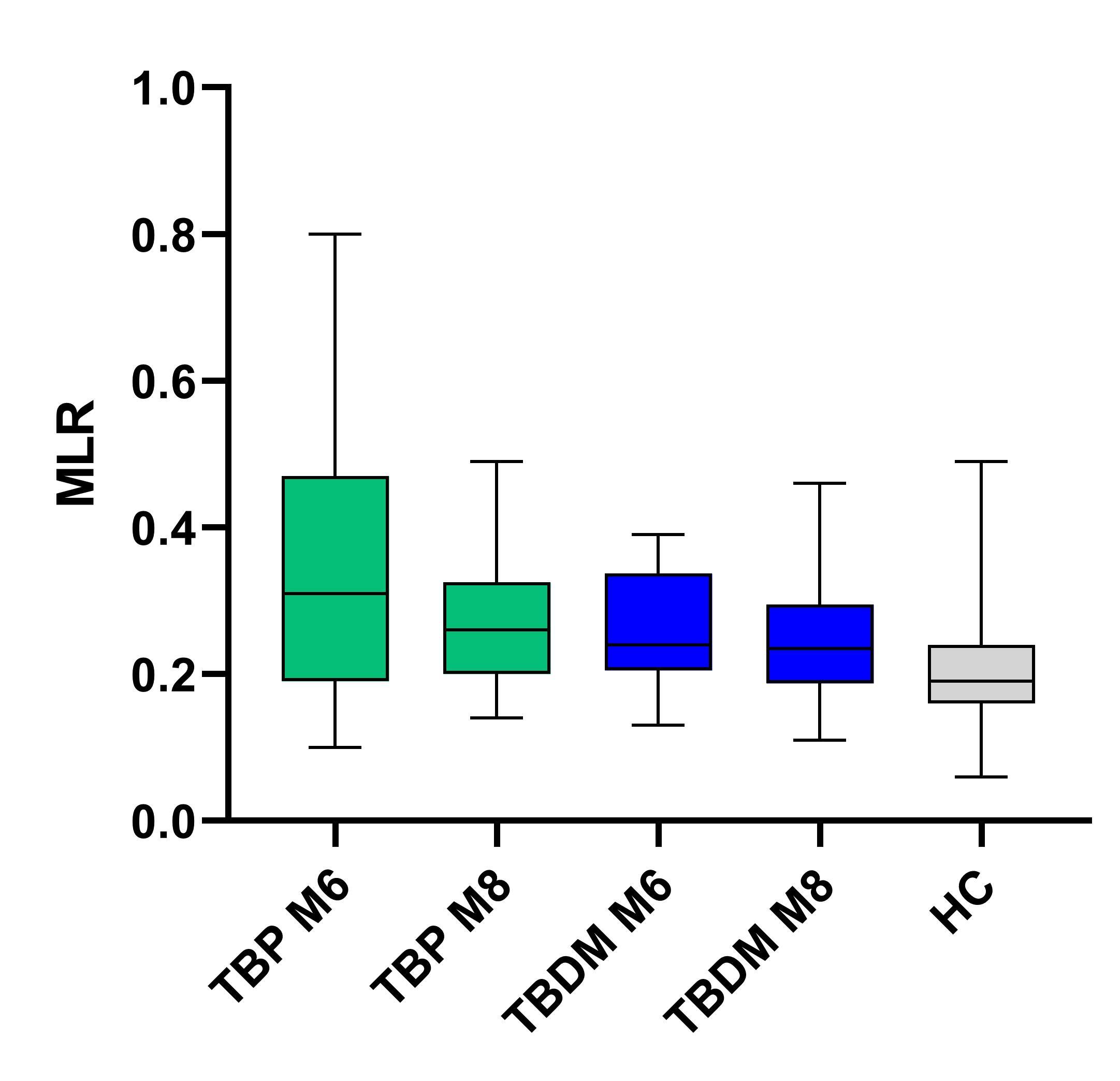

Supplement: Supplementary Figure 2 — Monocyte-to-lymphocyte ratio (MLR) levels of TBP and TBDM at M6 and M8 compared to healthy controls (HC). Box plots represent median and interquartile range. The upper and lower edges of the boxes represent the third and first quartiles (Q3 and Q1), respectively, while the line inside the box represents the median (Q2). [file Image2.jpeg]

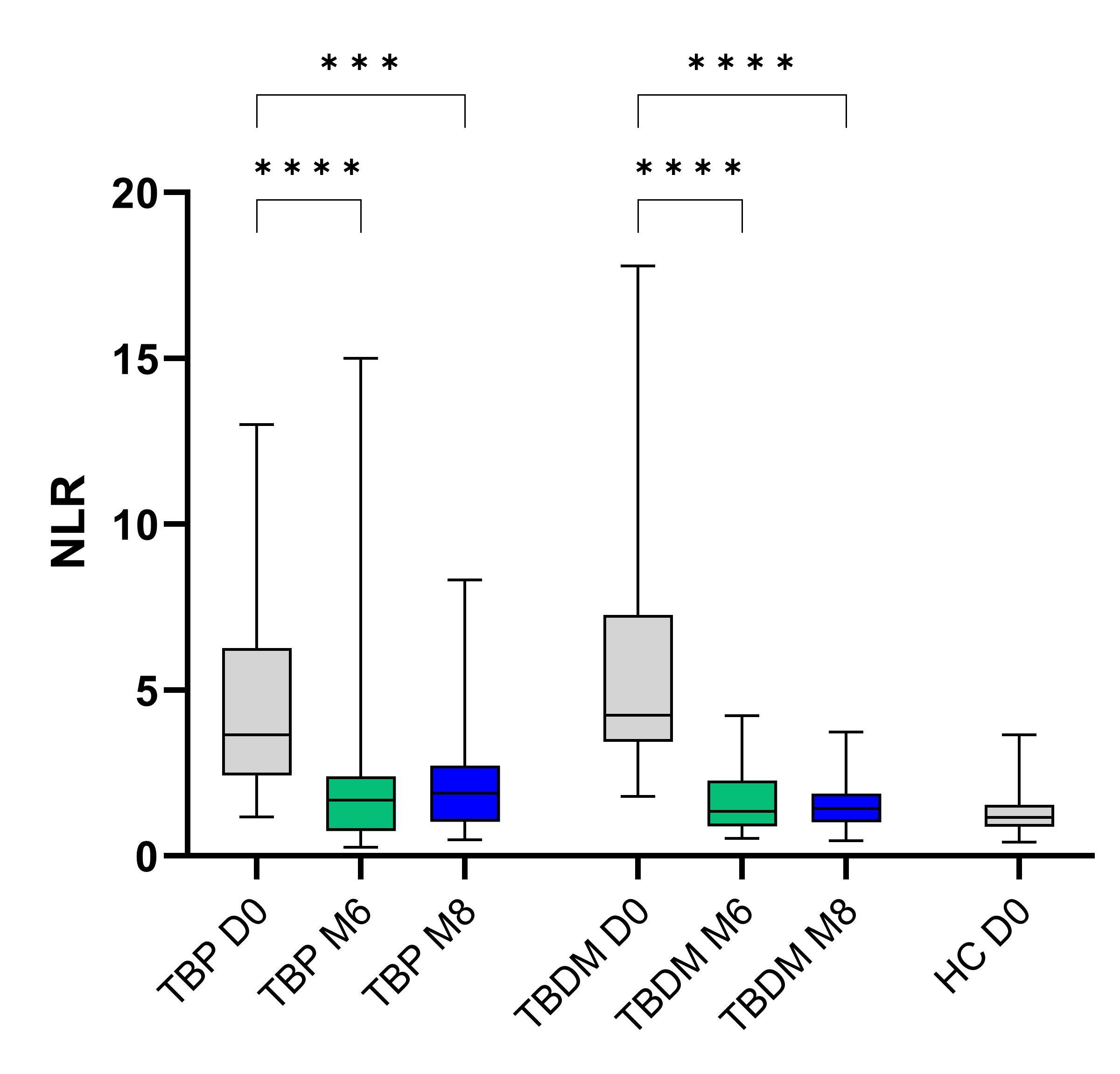

Supplement: Supplementary Figure 3 — Comparison of neutrophil-to-lymphocyte ratio (NLR) levels after TB treatment (M6 and M8) among the TBP and TBDM groups, and healthy controls (HC). Box plots represent median and interquartile range. The upper and lower edges of the boxes represent the third and first quartiles (Q3 and Q1), respectively, while the line inside the box represents the median (Q2). Data were analyzed using Kruskal-Wallis with Dunn’s multiple comparison test. ***: p<0.001, ****: p < 0.0001. [file Image3.jpeg]
